# Characterization of the complete chloroplast genome of *Peltoboykinia tellimoides* (Saxifragaceae)

**DOI:** 10.1080/23802359.2022.2070040

**Published:** 2022-05-03

**Authors:** Ranran Yang, Rui Li, Meifang Dong, Luxian Liu

**Affiliations:** Key laboratory of Plant Stress Biology, School of Life Sciences, Henan University, Kaifeng, China

**Keywords:** *Peltoboykinia tellimoides*, Saxifragaceae, chloroplast genome, phylogeny inference

## Abstract

*Peltoboykinia tellimoides*, which is distributed in Japan and China, is the type species of *Peltoboykinia*. In this study, we sequenced and assembled the complete chloroplast (cp) genome of *P. tellimoides* and reconstructed the phylogeny of Saxifragaceae based on the whole cp genome sequences. The cp genome of *P. tellimoides* was 156,274 bp in length, comprising a pair of inverted repeat regions (25,099 bp) separated by a large single copy region (88,109 bp) and a small single copy region (17,967 bp). The genome encoded 112 unique genes consisting of 78 different protein-coding genes, 30 transfer RNA and four ribosomal RNA genes, with 16 duplicated genes in the inverted repeats. Phylogenetic analysis indicated that *P. tellimoides* together with three *Chrysosplenium* species formed a high support clade, which was sister to *Micranthes melanocentra*.

*Peltoboykinia* (Engl.) Hara is a small genus of flowering plants belonging to the family Saxifragaceae and its native range is Southeastern China and Japan (Wu and Raven 2001). There are only two species within the genus, *P. tellimoides* (Maxim.) H. Hara 1937 and *P. watanabei* (Yatabe) H. Hara 1937, which are characterized by yellowish dentate petals 5 clothed with glandular, stout thick rhizome, glossy peltate leaves, inflorescentia cymoso-paniculata and hypanthium campanulatus (Hara 1937). *P. tellimoides* is the type species of this genus and the rhizome of the plant contained bergenin which is often found in Saxifragaceae (Nagai et al. 1969). However, the lack of research on the genetic background of *P. tellimoides* greatly affects the utilization of this species. Here, we generated genome skimming data for *P. tellimoides* to assembly the cp genome. The genome sequence of *P. tellimoides* was deposited into GenBank with the accession number MZ779205.

One *P. tellimoides* individual was collected from Suichang County, Zhejiang Province of China (118°52′56.69″E, 28°21′21.79″N). A specimen was deposited at the Herbarium of Zhejiang University (HZU; Pan Li, panli_zju@126.com) under the voucher number XXL170002-1, and no specific permissions were required for sample collection and the field study did not involve Endangered or protected species. Silica-dried leaf tissues were used to extract total genomic DNA by using Plant DNAzol Reagent (LifeFeng, Shanghai) according to the manufacturer’s protocol. The high-quality DNA was sheared (yielding ≤800 bp fragments) and the short-insert paired-end libraries preparation and sequencing were performed on Illumina X10 (Beijing Genomics Institute, Wuhan, China) with read length of 150 bp. The raw data was filtered by quality with Phred score <30 and all remaining sequences were assembled into contigs using the CLC de novo assembler beta 4.06 (CLC Inc., Rarhus, Denmark). Then the software Geneious R11 (Biomatters, Auckland, New Zealand) was used to reconstruct and annotated the cp genome with *Tiarella polyphylla* D. Don 1825 (GenBank accession number: MH708568) as a reference according to the description in Liu et al. (2017) and Liu et al. (2020). Maximum likelihood (ML) method was applied to reconstruct the phylogenetic tree for 22 whole cp genome sequences of Saxifragaceae species, with two Grossulariaceae species as outgroup. ML analysis was implemented in RAxML-HPC v8.2.12 on the CIPRES cluster (Miller et al. 2010) using the best-fit nucleotide substitution model (GTR + I + G) determined from jModelTest v2.1.4 (Posada 2008), and 1000 bootstrap iterations were conducted with other parameters using the default settings.

There were 35,589,288 paired-end reads generated from genome skimming sequencing for *P. tellimoides*, and 8,168,458 reads were removed from the raw data after trimming low quality sequences (Phred scores of 30 or less). The complete cp genome of *P. tellimoides* was 156,274 bp in length and shared the common feature of comprising two copies of IR (25,099 bp each) that divided the genome into two single-copy regions (LSC 88,109 bp; SSC 17,967 bp). The overall GC content of the total length, LSC, SSC, and IR regions was 37.9, 36.0, 32.7, and 43.0% respectively. Within the cp genome of *P. tellimoides* there were 112 unique genes, including 78 protein-coding genes, 30 tRNA genes, 4rRNA genes and 16 duplicated genes. Among them, six tRNA genes and nine protein coding genes contained a single intron, and *clpP* and *ycf3* contained two introns. The phylogeny showed that *P. tellimoides* and three *Chrysosplenium* species, including *C. flagelliferum* F. Schmidt 1868, *C. macrophyllum* Oliv. 1888, and *C. ramosum* Maxim. 1859, formed a highly supported clade, which in turn was sister to *Micranthes melanocentra* (Franch.) Losinsk. 1896 (Figure 1).

**Figure 1.**
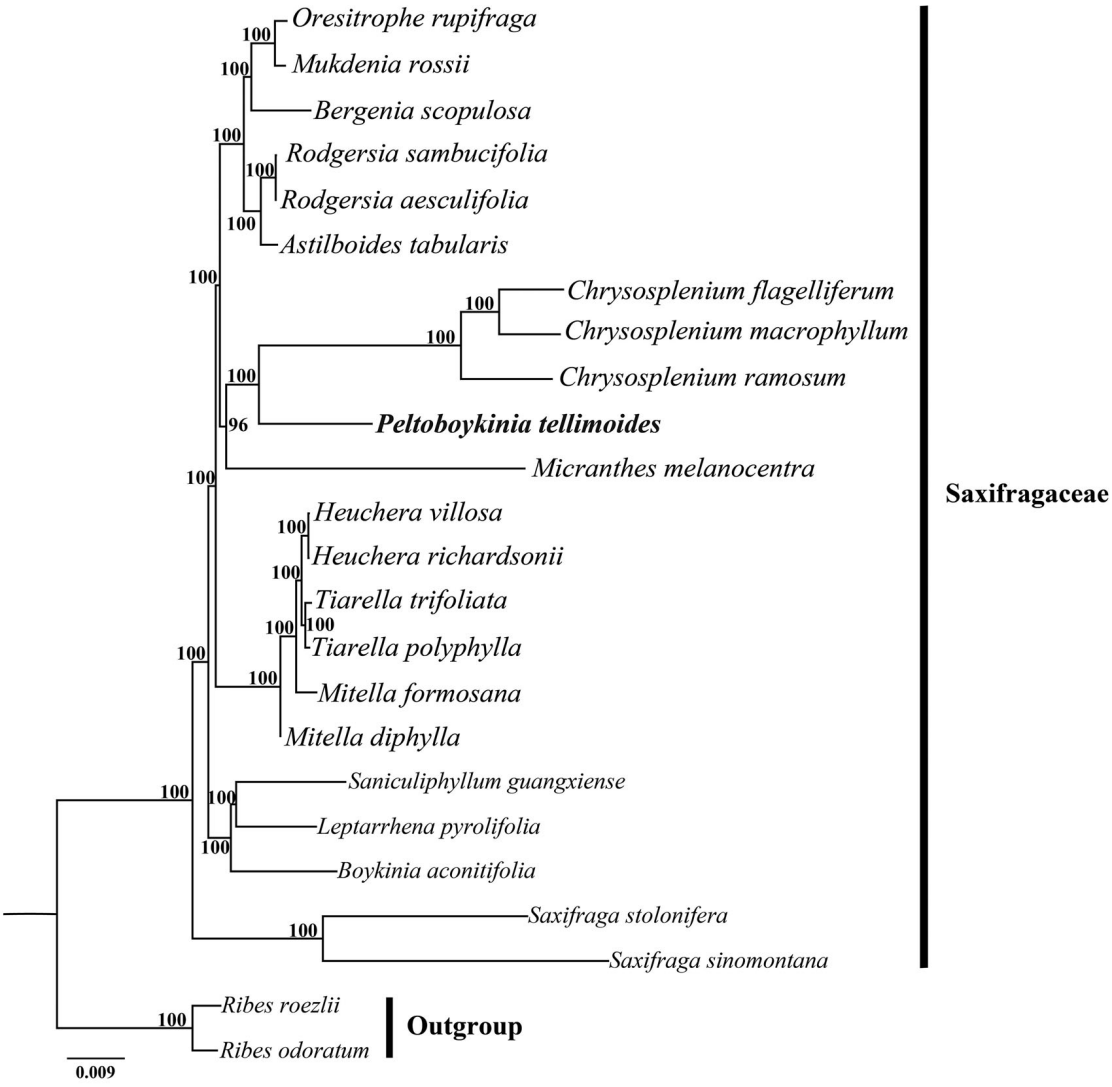
Phylogenetic tree reconstruction of 22 taxa of Saxifragaceae and two outgroups using ML method. Relative branch lengths are indicated at the top-left corner. Numbers above the branches represent bootstrap values from maximum-likelihood analyses. GenBank accession numbers of taxa are shown below, *Oresitrophe rupifraga* (MF774190), *Mukdenia rossii* (MG470844), *Bergenia scopulosa* (KY412195), *Rodgersia sambucifolia* (MN496077), *Rodgersia aesculifolia* (MW327540), *Astilboides tabularis* (MT316511), *Chrysosplenium flagelliferum* (MN729584), *Chrysosplenium macrophyllum* (MK973001), *Chrysosplenium ramosum* (MK973002), *Peltoboykinia tellimoides* (MZ779205), *Micranthes melanocentra* (MT740256), *Heuchera villosa* (MH708563), *Heuchera richardsonii* (MH708562), *Tiarella trifoliata* (MH708572), *Tiarella polyphylla* (MH708568), *Mitella formosana* (MH708565), *Mitella diphylla* (MH708564), *Saniculiphyllum guangxiense* (MN496078), *Leptarrhena pyrolifolia* (MN496070), *Boykinia aconitifolia* (MN496058), *Saxifraga stolonifera* (MH191389), *Saxifraga sinomontana* (MN104589), *Ribes roezlii* (MN496076), *Ribes odoratum* (MT081309).

## Data Availability

The genome sequence data that support the findings of this study are openly available in GenBank of NCBI at https://www.ncbi.nlm.nih.gov/ under the accession no. MZ779205. The associated Bio-Project, SRA and Bio-Sample numbers of the raw sequence data for assembling the cp genome are PRJNA754512, SRR15460330, and SAMN20792568, respectively.
